# Transcription of the T4 late genes

**DOI:** 10.1186/1743-422X-7-288

**Published:** 2010-10-28

**Authors:** E Peter Geiduschek, George A Kassavetis

**Affiliations:** 1 Division of Biological Sciences, Section of Molecular Biology, University of California, San Diego, La Jolla, CA 92093-0634, USA

## Abstract

This article reviews the current state of understanding of the regulated transcription of the bacteriophage T4 late genes, with a focus on the underlying biochemical mechanisms, which turn out to be unique to the T4-related family of phages or significantly different from other bacterial systems. The activator of T4 late transcription is the *g*ene 45 *p*rotein (gp45), the sliding clamp of the T4 replisome. Gp45 becomes topologically linked to DNA through the action of its clamp-loader, but it is not site-specifically DNA-bound, as other transcriptional activators are. Gp45 facilitates RNA polymerase recruitment to late promoters by interacting with two phage-encoded polymerase subunits: gp33, the co-activator of T4 late transcription; and gp55, the T4 late promoter recognition protein. The emphasis of this account is on the sites and mechanisms of actions of these three proteins, and on their roles in the formation of transcription-ready open T4 late promoter complexes.

## Introduction

T4 late genes are transcribed from simple promoters consisting of an 8-base pair TATA box placed ~1 helical DNA turn upstream of the transcriptional start site (the location of the bacterial σ^70^-family RNA polymerase (RNAP) promoter -10 site). A significant AT base pair preponderance characterizes the segment immediately downstream of the TATA box that strand-separates when the late promoter opens for initiation of transcription; there is no sequence conservation at the position corresponding to the bacterial promoter -35 site.

**Figure 1:**
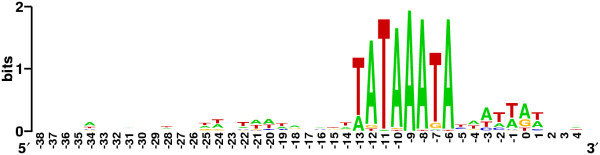
The T4 late promoter sequence logo.

Fifty of these sites are listed for the T4 genome [1,2]. The consensus first proposed by Christensen and Young [3] is tightly adhered to overall (Figure 1), perfectly so at 32 sites, with A(-13) in place of T at nine sites and other single deviations from consensus at the remaining sites, with two exceptions, (one a TA→AT change). Variant T4 late promoters are used for (basal) transcription *in vitro *[4] and a number of variant promoters have also been associated with RNA 5" ends *in vivo *[5,6] (Three cautionary notes: 1) these 50 sites have not all been identified as promoters that are active *in vivo*; 2) some of the RNA 5" ends that have been mapped to putative promoters were specified by primer extension analysis, which does not distinguish between 5" ends generated by bona fide initiation and endonucleolytic processing; 3) the relative rates of initiation at consensus and variant T4 late promoters *in vivo *have not been determined.) While all early and middle transcripts have the same polarity, that is, counterclockwise in the standard representation of the T4 genetic map, and complementary to the DNA *l *strand [7], late transcripts have either polarity. At several sites, both T4 DNA strands are transcribed at different times of the multiplication cycle [8,9].

Transcription initiating at these simple promoters requires the function of T4 genes 33 and 55. These two genes hold a special place in the history of molecular biology, because they are the first master regulators of a developmental program of gene expression to have been discovered [10]. Both genes encode RNAP-binding proteins [11,12]: the gene 55 protein (gp55) is the smallest and one of the most highly divergent members of the σ^70 ^family [13-15], while gp33 has no recognizable homology with σ proteins. The phenotypes of cells infected with 33^- ^and 55^- ^phage are, however, not the same. In the absence of gene 55 function, late genes are not transcribed. In contrast, some late transcription eventually materializes, and late proteins are also made at reduced levels, in cells infected with gene 33-defective phage. These differences of phenotype of gene 33 and gene 55 mutants reflect the different mechanisms of action of gp33 and gp55 in transcription, as discussed below. Late transcription normally also requires DNA replication [10,16] and is, in fact, coupled to concurrent DNA synthesis [17].

The coupling of late transcription to DNA replication is enforced by the action of gp30, the T4 DNA ligase [18]. Single-strand breaks make T4 DNA subject to nucleolytic attack, but protecting against that degradation by knocking out the exonuclease function encoded by gene 46 generates a situation in which late transcription occurs in the absence of DNA replication (e.g., in the absence of T4 DNA polymerase (gp43) function) [19,20]. Thus, the just-specified gene 30^-^/43^-^/46^- ^triple mutant serves as a platform for finding proteins that are not only required for T4 DNA replication but have an additional direct role in late transcription. Those experiments clearly identify the involvement of gp45, the sliding clamp processivity factor of the T4 DNA polymerase holoenzyme, in T4 late transcription [21]. (That this approach does not equally clearly identify the involvement of the gp44/62 clamp loader complex in T4 late transcription is puzzling, as discussed further on.)

In summary, the primary direct roles in T4 late transcription are played by three proteins--gp55, gp33 and gp45--and by a transient form of the T4 DNA template that is generated in the process of replication. The focus of the rest of this account is on explaining the mechanisms of action of these components.

### Gp55

**Figure 2:**
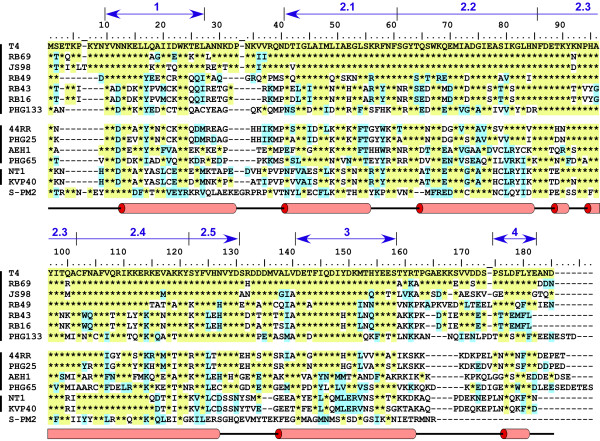
**Amino acid sequence conservation of gp55. **All T4-related phage genomes sequenced to date (see [59], which is a review by Petrov, et al., in this series) contain readily identifiable gp55 homologues [81]. Four segments of sequence conservation can be noted. The central and largest segment 2 allows the distant relationship to domain 2 of σ^70 ^to be discerned, primarily through correspondence with σ^70 ^conserved segments 2.1 and 2.2 and secondary structure. The presumption that segment 2.4 harbors the late promoter recognition element of gp55 is speculative. Conserved segment 4 is the sliding clamp-binding epitope. Conserved segments 1 and 3 share no recognizable sequence similarity with σ^70^. Whether they correspond functionally with σ segment 1.1/1.2 and 3.1, respectively, is not known. The numbering of residues is continuous for the T4 protein. Amino acid sequences of the T4, RB14 and RB32 proteins are identical; only T4 is listed. RB49 and phi-1 gp55 are also identical except for Q30 (RB49)→E30 (phi-1); only RB49 is listed. A secondary structure prediction from HHpred, with α-helices as cylinders, is shown below the alignment. Vertical lines at the side cluster phages infecting (top to bottom): *E. coli *(133 was isolated as an *Acinetobacter *phage); *Aeromonas *species; and *Vibrio *species. The more divergent S-PM2 protein is the only representative of the completely sequenced cyanobacterial phages that has been included for this presentation. (The cyanobacterial RNAPs constitute a separate clade in the phylogeny of the multisubunit enzymes, as do the archaeal RNAPs and the individual eukaryotic nuclear RNAPs I-V.)

**Figure 3:**
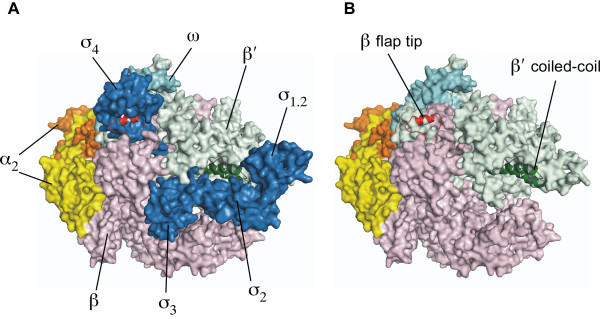
**Bacterial RNAP holoenzyme. A**. The *Thermus aquaticus *RNAP holoenzyme. The β (pink), β" (pale green), α_2 _(yellow, orange; without their C-terminal domains) and ω (cyan) subunits are identified, and the β subunit flap (red), which is the attachment site of σ domain 4 and gp33, as well as the β" coiled-coil (green), which is the docking site of σ domain 2 and gp55, are emphasized. σ domains 1.2, 2, 3 and 4 (dark blue) are identified. **B**. The same, with σ removed (i.e., RNAP core, but with the coordinates of the holoenzyme) (Adapted from [26]).

Gp55 is a very small, highly diverged σ^70^-family protein (Figure 2). The σ^70^/σ^A ^subunits of the bacterial RNAPs comprise 4 globular domains (σ_1_, σ_2_, σ_3 _and σ_4_; Figure 3) that are widely separated on the surface of the RNAP holoenzymes. When σ detaches from the RNAP core, these domains swap their sites of interaction with the β and β" RNAP subunits for internal contacts and assume a compact structure [22,23]. The σ structural domains also correspond with segments of sequence conservation (segments 1.1, 1.2; 2.1-2.4; 2.5 and 3.1; 4.1 and 4.2;[15]). Discernible similarity of gp55 with σ^70 ^is confined to domain 2 [13-15], which provides the principal RNAP core-binding and -10 DNA site-recognition functions of σ proteins (involving conserved sequence segments 2.2 and 2.4, respectively) [24-26]. Since a direct determination of gp55 structure is not yet at hand, what follows pieces together the information that can be derived from site-directed mutagenesis, analysis of function and interactions *in vitro, *and consideration of amino acid sequence conservation.

Gp55 is the promoter recognition subunit of the T4 late gene-transcribing RNAP holoenzyme [27] and confers the ability to execute basal level accurately initiating transcription on unmodified and exhaustively σ-stripped *E. coli *RNAP core. This basal transcription by gp55•RNAP is sensitive to ionic strength, and greatly reduced at lower temperature or when relaxed DNA is used as template in place of supercoiled plasmid DNA [27-31].

Initial binding of gp55•RNAP to DNA is not highly specific, in the sense that it does not greatly favor promoters relative to non-promoter sequence. (What this means operationally is that, for example, DNase I footprints of initially forming closed T4 late promoter complexes are not discernible above the background of non-specific DNA binding under conditions that are satisfactory for analysis of closed σ^70^•RNAP promoter complexes) [32,33]. In contrast, open T4 late promoter complexes are site-specific, stable and readily detected by footprinting [32,34]. The acquisition of additional sequence discrimination on promoter opening implies sequence-specific recognition of some feature of the open promoter (perhaps its separated non-transcribed DNA strand) by gp55, but this has not been demonstrated directly.

The σ segment 2.2-equivalent RNAP core-binding motif of gp55 has been inferred on the basis of alanine-scan mutants analyzed for RNAP core-binding, basal and activated transcription [35]. This segment of gp55 is highly conserved (Figure 2). Extension of the alignment and secondary structure prediction suggests that residues ~42-122 constitute the σ_2_-equivalent domain of gp55. Conservation of sequence among gp55 homologues extends outside this segment (Figure 2). In particular, absolute conservation of aromatic residues at N-proximal positions 10 and 23 of segment 1 is notable, as is conservation of sequence for residues ~141 - 156 (segment 3; numbering refers to the T4 protein) implying essential gp55 functions that might be related to σ^70 ^segments 1.1 and 3.1, respectively. Sequence of a short hydrophobic and acidic C-terminal segment of gp55 is also conserved. This is the sliding clamp-binding epitope of T4 gp55 [36,37] and its conservation suggests that ability of the late gene-transcribing RNAP holoenzyme to bind the sliding clamp is a widely shared function of T4-related family phages. A 17-residue segment connecting the C-terminal epitope of gp55 to the rest of the protein is highly divergent in sequence and of varying length even among phages infecting *E. coli*. In the case of the T4 protein, extensive amino acid substitutions as well as insertions of a flexible (Ser-Gly) linker and small deletions do not eliminate the ability to support sliding clamp-activated late transcription [33]. This gp55 segment may be an unstructured linker connecting the sliding clamp-interacting C-terminus with the RNAP core-bound rest of the protein, somewhat comparable with the flexible linker that connects the N- and C-terminal domains of the RNAP α subunits [38].

### Gp33

The 112-residue gp33 binds to the flap tip of the RNAP β subunit [39]. This is also the RNAP core attachment site of σ domain 4, which recognizes the -35 promoter element. Thus, gp33 can be thought of as a σ_4 _mimic, and gp55 together with gp33 as a split σ. On the other hand, the β flap, which juts out over the RNA exit pore of the elongating transcription complex, is also the attachment site of other effectors of transcription, notably the phage λQ protein and other regulators of transcriptional elongation and termination. Moreover, gp33 does not recognize DNA sequence (and no sequence recognition is required since T4 late promoters do not have an upstream/-35 element). Instead, gp33 represses basal transcription [40,41] by diminishing promoter as well as general non-specific DNA binding. Binding of RNAP to DNA ends and DNA end-initiating transcription is exempt from this inhibition [42].

**Figure 4:**
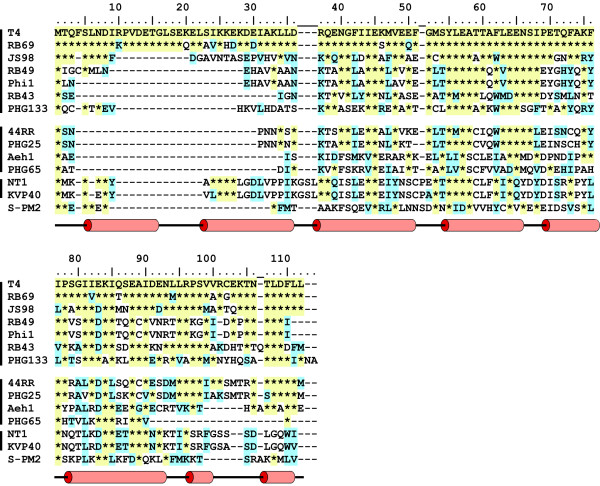
**The limited sequence conservation of gp33. **The presentation of the sequence alignment follows Figure 2. Amino acid sequences of the T4 and RB14 proteins are identical; RB32 gp33 differs only by E50→K; only the T4 protein is listed. RB43 and RB16 gp33 are identical and only RB43 is listed. A secondary structure prediction from HHpred is shown below the alignment.

Conservation of amino acid sequence among gp33 homologues is primarily confined to individual residues in the C-terminal two-thirds of these proteins, which include the RNAP core binding site and the C-terminal sliding clamp-binding epitope (Figure 4). A recently completed determination of the structure of a gp33 complex with the *E. coli *RNAP β flap [43] and modeling into the *Thermus *RNAP structures [24,25] accounts for this conservation in terms of protein-protein contacts in this complex, suggests additional gp33:RNAP core interactions [43] and rationalizes extensive mutational analysis of gp33:RNAP binding and function [33,39]. The N-proximal one-third of gp33 is highly variable, entirely missing in homologues from other *E. coli*-infecting T4-related phages. There is no discernible similarity of amino acid sequence between gp33 and σ proteins, but the new structure allows functional correspondences between individual gp33 and σ^70 ^domain 4 residues to be seen.

It has been proposed that when it binds to the β flap, gp33 occludes a non-specific DNA-binding site on RNAP core, that this RNAP core site also interacts non-specifically with DNA upstream of the T4 late promoter's -10 element and, in so doing, contributes to the promoter affinity of gp55•RNAP without contributing to selectivity [42]. The exemption of DNA-end-initiating transcription from inhibition by gp33 is presumed to be a direct consequence of its mechanism: binding to, and initiating transcription at, linear DNA template ends involves threading those ends through the downstream DNA channel for access to the catalytic center of RNAP, out of contact with β flap-bound gp33 and the upstream-facing part of RNAP.

### Gp45

Gp45 is the T4 representative of the sliding clamp proteins. Sliding clamps are six-domain rings with a central hole large enough to accommodate a DNA helix: head-to-tail homodimers of 3-domain subunits in the case of the *E. coli *replisome's β protein; homotrimers of 2-domain subunits in the case of gp45 and the eukaryotic PCNA (*p*roliferating *c*ell *n*uclear *a*ntigen); homo- or heterotrimers of 2-domain subunits in the case of archaeal PCNA (for a review, see [44] and [45], which is an article by Mueser, et al., in this series). α-helices with a net positive charge line the central cavity and antiparallel β sheets with a net negative charge form the periphery of sliding clamps. Pseudo-6-fold symmetry axes run through the centers of the sliding clamps, except for the case of gp45, whose C-proximal domain of each protomer is somewhat shorter than the N-proximal domain, generating a form that is closer to triangular than hexagonal (i.e., with 3-fold symmetry instead of 6-fold pseudo-symmetry) [46,47].

The lateral faces of the sliding clamp are chemically distinctive; notably, the lateral face with the protruding C-terminus presents a hydrophobic patch on each protomer that serves as a binding site for the numerous and functionally diverse ligands that sliding clamps tether to DNA. (The sliding clamps are, for that reason, also aptly referred to as sliding toolbelts.) The ligands of the T4 sliding clamp include its clamp loader, the gp44/62 complex, and the highly similar hydrophobic and acidic C-termini of gp43, gp55 and gp33. For gp43, this interaction establishes processive DNA chain elongation (by confining DNA polymerase to the one-dimensional space of the DNA thread (see [45], by Mueser, et al., this series).

Crystal structures of sliding clamps show them all as closed rings. In contrast, detailed analysis shows that the gp45 trimer in solution is open at one monomer interface and out of plane, somewhat like a split-ring lock washer [48]. All sliding clamps require loading factors that mount them on to DNA at double-strand-single-strand/primer-template junctions in an ATP hydrolysis-requiring process. The gp44/62 complex is the T4 clamp loader and it also loads gp45 on to DNA at nicks. Since their lateral faces are not identical, there are two distinguishable orientations of sliding clamps on DNA. The DNA strand with the 3"OH end determines the orientation of the clamp loader and, in turn, of the loaded sliding clamp. Thus, in the case of clamp loading at a DNA nick, for example, switching the strand that is interrupted reverses the orientation of the sliding clamp on DNA and therefore the polarity of its protein interactions. The same face of gp45 that attaches to the clamp loader also binds gp43 and, as argued below, the gp55- and gp33-containing T4 late RNAP holoenzyme.

The RB69 sliding clamp (81% identity of amino acid sequence with the T4 protein) has been co-crystallized with its ligand, the 11 C-terminal residues of the DNA polymerase [47]. The structure of the complex shows attachment of the hydrophobic 11-mer to the already referred to hydrophobic patch on the gp45 face with the protruding C-end of the protein (the C face), with only one of the three available sites occupied in each gp45 trimer. This is also the ligand-interaction mode of other sliding clamps [44,47,49]. In contrast, the preferred binding site of the C-terminal epitope of T4 gp43 in solution is the open gp45 inter-monomer interface [50]. (The gp45 ring being closed in crystals, that site would not be available for complex formation.) Thus, at least two different attachment sites on gp45 are apparently available for its gp43, gp55, gp33 and clamp loader partners. These sites do not offer the same affinity to their ligands, but they may both play roles in clamp loading, replication and/or transcription.

Gp45 sliding along DNA can be detected by DNA-protein photochemical cross-linking as occupancy of interior DNA sites that is dependent on a DNA-loading site, a clamp loader and ATP [51]. Experiments of that type show that gp55 tracks along DNA as a gp45 ligand [52]. This implies an ability of the sliding clamp to confer a mode of promoter searching that is dominated by processive one-dimensional scanning along the DNA thread. A snakes-and-ladders game model has dominated thinking about how proteins find their sites on genomes [53]. Sliding clamp-facilitated promoter searching is more-snakes-less-ladders. Whether facilitating promoter searching increases transcriptional activity depends on whether it is rate-limiting. This is unlikely to be the case for basal (gp33-independent) transcription, for which promoter opening is slow, as described below, but is not excluded for activated transcription, which is marked by very rapid promoter opening [32].

T4 sliding clamps must be loaded onto DNA by their clamp loaders in order to execute their functions in DNA replication and transcription. It is puzzling, therefore, that gene 44 and 62 *amber *mutations are clearly and nearly absolutely replication-defective (D0 phenotype) [10], but that the requirement for gp44/62 complex function in T4 late transcription was not clearly identified by the analysis that established the essential role of gp45 [21]. As referred to below, macromolecular crowding agents, such as poly(ethyleneglycol), allow gp45 to escape total reliance on the clamp loader for activating DNA replication by gp43 and T4 late transcription [54,55]. The bacterial cytoplasm is a macromolecularly crowded medium, suggesting that these observations may have some physiological relevance, but they do not account for differences of effect of clamp-loader mutations on replication and late transcription [21]. The explanation of these differences may instead reside in the existence of additional interactions of the T4 clamp loader with the T4 replisome.

### Other genes and functions

The T4 genome encodes more than 300 proteins, many with unknown or barely explored function. Several of these genes and functions relate to viral transcription and they have been most recently referred to in the detailed 2003 overview of the T4 genome [2]. As pointed out there, the functions of most of these proteins probably relate to early and middle viral transcription (see [56], which is a review by Hinton in this series) and to shutting off host transcription under conditions (such as nutrient limitation and stress) that are very different from those that were used for the classical analysis of the T4 multiplication cycle in early log phase cells. There is nothing new regarding them to report in the context of this chapter, with the possible exception of DsbA. *dsbA, *which first came to attention as the immediately upstream-lying and translationally coupled ORF to gene 33 [40], encodes an ~10 kDa DNA-binding protein, for which specific A/T rich DNA-binding sites overlapping two late promoters were identified but with surprisingly low affinity (in the μM range for K_d _at moderate ionic strength) [57,58]. Finding *dsbA *to be a non-essential gene [2] has not encouraged further analysis in the T4 late transcription *in vitro *system, but genome sequencing in the T4-related phage family (see [59], which is a review by Petrov, et al., in this series) brings an interesting feature of *dsbA *to light. As already mentioned, the N-terminal 1/3 of gp33 is highly divergent among T4-related phages; even homologues from phage that are all capable of infecting *E. coli *lack the N-terminal 20-30 codons of the T4 protein. Nevertheless, *dsbA *genes are widely distributed and the *dsbA*-gene 33 ORF overlap, indicating translational coupling, is conserved, suggesting a significant role for *dsbA*, possibly relating to gene 33 and late transcription, that remains to be discovered. Our tentative examination of this issue has not been encouraging: under the standard conditions of the *in vitro *transcription system [32,33] no effect of DsbA on gp33-repressed or gp33/sliding clamp-activated transcription was discerned (V. Jain, unpublished observation).

### The mechanism of activation

The 8-bp T4 late promoter resembles σ^70 ^extended -10 promoters in that DNA sequence recognition is confined to the downstream site at which promoter opening is initiated and proceeds in the absence of a σ_4_-equivalent domain (in the case of the T4 late RNAP) and without requiring σ_4 _participation (in the case of σ^70^•RNAP). Gp55 dictates specifically initiating transcription at late promoters by unmodified *E. coli *RNAP core (RNAP^U^) and by the T4-modified core enzyme (RNAP^T4^), whose α subunits are ADP-ribosylated in both C-terminal domains (CTD) at Arg265. As already mentioned, transcription is more active on supercoiled than on relaxed (nicked circular or linear) DNA, at higher temperature and at lower ionic strength [27-31], generally in keeping with the activities of most weak bacterial promoters. Kinetic analysis of transcriptional initiation by gp55•RNAP^T4 ^at the consensus gene 23 promoter in linear DNA (limited to a single temperature and in a single reaction medium) indicates weak promoter binding and relatively slow promoter opening [32].

Promoter opening by σ^70 ^family RNAPs is temperature-dependent, to a significant degree adjusted to bacterial lifestyle in the sense that it operates at higher temperature in thermophiles than in mesophiles [60], and it is a reversible process [61,62]: when the λP_R _and *gal *P1 promoters (to take one example each of a strong -35/-10 promoter and an extended -10 promoter) are opened at 37°C and brought to 0°C they close (although that process can be relatively slow, implying the existence of a significant kinetic barrier). In contrast, the T4 late promoter opens thermo-irreversibly: while it does not open at 0°C (even on a multi-hour time scale) it does not close at 0°C once it has been opened at higher temperature. The kinetic block has been suggested to lie on the promoter-closing pathway [63].

Activated transcription requires the participation of DNA-mounted gp45 and RNAP-bound gp33. The critical observations leading to the current understanding of activated transcription were made with an *in vitro *system that was designed to allow concurrent leading-strand DNA synthesis and late transcription, using a plasmid DNA template with a uniquely placed single-strand break serving as the initiation site for DNA synthesis. It was relatively promptly found that transcriptional activation in this *in vitro *system does not require DNA replication but does require the participation of three T4 replication proteins, the gp44/62 complex and gp45, ATP or dATP hydrolysis (ATP-γ-S, the very slowly hydrolyzing ATP analog blocking activation), and RNAP from T4-infected cells. Activation is not supported by gp55•RNAP^U^, and absolutely requires gp33 [41].

The DNA template's single-strand break, which is essential for transcriptional activation, has the properties of an enhancer in that it can be placed close to, or at kbp separation from the promoter, but with the special constraint that the DNA break has to be in the non-transcribed strand of the activated promoter, so that switching the nicked strand switches the polarity of transcriptional activation [30]. The general mode of action of the enhancer was established by showing that it acts strictly *in cis *and that it requires a continuous, unobstructed path to the promoter [64]. The gp44/62 complex having been established as the non-processive DNA-loading factor for gp45 at about the same time [65-68], and DNA nicks being candidate loading sites for gp45, it was probable at this point [64] that the required continuous DNA path allows gp45 to slide from its DNA-loading site to the promoter. That this is the case was established by showing that gp45 becomes a stably bound part of the activated promoter complex, and is located at its upstream end [34], tethered there by the C-termini of gp55 and gp33 [36], as already mentioned.

Loading gp45 onto DNA at nicks does not require the gp32 single-stranded DNA-binding protein. However, primer-template junctions are more efficient gp45-loading sites in the presence of gp32 than are DNA nicks. The transcription-activating primer-template junction also has a polarity constraint: it must be located downstream of its target promoter [69]. The existence of this constraint establishes that the same lateral face of the sliding clamp interacts with T4 DNAP and with the late gene-transcribing gp55•gp33•RNAP holoenzyme. In contrast, the DNA-nick gp45-loading site can be located upstream or downstream of its target promoter [64]. This is a reflection of the ability of the gp45 clamp to slide across a DNA break, whereas it does not slide efficiently across single-stranded DNA [69]. In the presence of macromolecular crowding agents such as high molecular weight poly(ethyleneglycol) (PEG), gp45 can activate transcription and replication in the absence of the clamp loader [54,55]. Activation under these conditions also dispenses with the need for a nick or primer-template loading site as well as ATP hydrolysis, and functions with relaxed closed circular as well as blunt-end linear DNA. The requirement for gp33 and gp55 is retained. Needless to say, this finding also establishes gp45 as the activator of late transcription [55].

**Figure 5:**
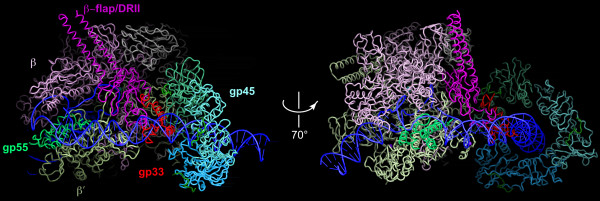
**A composite partial molecular model of the sliding clamp docking on an RNAP:promoter complex. **The structure of the RB69 sliding clamp [47] has been docked against a *Taq *RNAP holoenzyme fork junction promoter DNA complex [25]. Evidence from site-specific DNA-protein photochemical cross-linking and DNA footprinting [34] specifies that the sliding clamp abuts RNAP. Gp33 is placed in the model in accordance with the recent determination of its structure in complex with the *E. coli *β flap and DRII (amino acids 831-1057) by K-A.F. Twist and S.A. Darst [43][K-A.F. Twist, P. Deighan, S. Nechaev, A. Hochschild, E.P. Geiduschek & S.A. Darst, in preparation] and a complete structural model of *E. coli *RNAP based on a combination of approaches [82]. Placement of the C-end of gp33 in proximity to DNA is consistent with evidence from site-specific DNA-protein cross-linking [34]. The rotational orientation of gp45 is arbitrary, but is likely to be constrained by the interacting RNAP surface and also by the short tether to gp33. The location of the C-end of gp33 on the sliding clamp in the T4 late promoter complex is not known; a C-terminal 11-mer of phage RB69 DNA polymerase from the structure in [47] has not been removed and is barely visible, but its relevance to the late promoter complex is unclear, as discussed in the text. Residues 44-123 of gp55, comprising its RNAP core- and DNA-biding sites, have been modeled based on homology with σ^70 ^domain 2 [26] and docked onto the β" subunit coiled-coil. Colors of components are indicated in the Figure. (Images provided by K.-A. Twist and S.A. Darst and reproduced with their permission.)

These facts about the sliding clamp-activated T4 late promoter complex suffice for the construction of a composite partial molecular model (Figure 5) based on the structure of the *Thermus aquaticus *(*Taq*) RNAP-fork junction complex [26], the just-recently determined structure of gp33 in complex with the β subunit flap domain and ~100-residue dispensable region (DR)II of *E. coli *RNAP [43], and gp45 [46]. The DNase I footprint of the activated and basal open promoter complexes differ by a 13 bp extension at the upstream end, almost exactly the DNA span of the sliding clamp (see also [70]). Thus, the sliding clamp must be pressed close to RNAP core on DNA, with the α subunit C-terminal domains pushed out of the way. The only segment of gp55 that is represented in Figure 5 is region *2.1-2.4 *(amino acids *44-123*, Figure 2, modeled by homology with *Taq *σ^70 ^domain 2 [26]) attached to the β" subunit coiled-coil. The model is consistent with gp33 (presumably in a C-proximal segment) lying within cross-linking proximity of DNA (~1 nm) at bp -39 and -36/-35 of the activated T4 late promoter complex [71], although it does not bind sequence-specifically to it.

**Figure 6:**
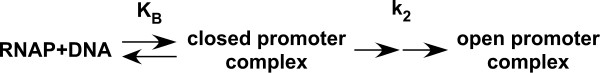
A simplified 2-step model for kinetic analysis of the formation of initiation-ready open promoter complexes.

The functional consequences of attachment of the sliding clamp to the upstream end of RNAP in the activated late promoter complex through its interactions with hydrophobic and acidic motifs at the C-termini of gp55 and gp33 are a greatly increased overall rate of promoter opening. Kinetic analysis within a simplified 2-step framework for bacterial promoters [61,62,72] (Figure 6) indicates that the sliding clamp increases the effective affinity of the initially forming closed promoter complex (K_B_) and the phenomenological first order rate constant for the subsequent step(s) of promoter opening (k_2_) for a combined ~300-fold activation (measured at 30°C, with RNAP^T4^) [32]. Basal transcription is repressed about one order of magnitude by gp33 (e.g., [42]); relative to this lowest activity of the gp33•gp55•RNAP^T4 ^holoenzyme, the sliding clamp mediates a >1,000-fold activation [32] (*Footnote *1, which is embedded in the text below). The notion that tethering the promoter complex to DNA would increase its effective affinity is intuitively uncomplicated; that gp45 also lowers the activation energy barrier for promoter opening by holding on to gp55 and gp33 is less so; what follows suggests that this effect is probably mediated by gp33. Changes of promoter activity of this magnitude generate the emergence of qualitatively new properties. For example, avid association of the gp45-activated RNAP complex with DNA allows open promoter complexes to form in competition with high concentrations of the polyanionic competitor heparin [33].

(*Footnote *1, A technical note: the above kinetic scheme adequately describes basal transcription with its characteristically slow promoter opening, and serves to parametrize a simple kinetic analysis of the just-cited work [32]. The principal result of that analysis--that the activator increases the second order rate constant for forming the open promoter complex by several hundred-fold relative to basal transcription and even more relative to gp33-repressed transcription, and that this increase results from a combination of tighter promoter binding and faster promoter opening--is not in question. However, the kinetic scheme is probably an inadequate representation of gp45-activated transcription, which is characterized by very rapid promoter opening and low selectivity, so that formation of the closed but precisely positioned promoter complex may not come to equilibrium.)

The highly similar C-terminal sliding clamp-binding motifs of gp55, gp33 and DNA polymerase (gp43) can be freely interchanged; replacing both C-terminal motifs of gp55 and gp33 with the C-terminal motif of gp43 leaves transcriptional activation *in vitro *quantitatively unchanged [33]. While this eliminates the possibility that their C-ends direct gp55 and gp33 to different binding sites on gp45, it does not settle the question of where, on the sliding clamp, these sites are located. The open interface of the gp45 trimer is the preferred binding site of gp43; while a sliding clamp cannot be simultaneously open at two sites, binding by both the gp55 and gp33 termini to separate clamp subunit interfaces is conceivable if at least one ligand seals its opening. Alternatively, even identical C-terminal motifs might occupy non-identical binding sites on gp45 (e.g., one ligand inserted into a monomer interface and the other attached to a lateral face hydrophobic patch) under the steric constraint that is imposed by gp33 and gp55 attachment to RNAP core.

The sliding clamp activator is held by two "arms" that extend from the gp33•gp55•RNAP. Separately detaching each of these arms has drastically different consequences for transcriptional activation: gp33:clamp binding is absolutely essential, while eliminating gp55-binding reduces but does not eliminate activation [33,36]. Conversely, gp45 exerts little or no activating effect on basal transcription by gp55•RNAP (*Footnote *2, which is embedded in the text below). "One-armed" partial activation of transcription by gp45 (i.e., in the absence of the gp45:gp55 interaction) is also sensitive to inhibition by heparin [33]. This probably reflects a loss of late promoter binding affinity (K_B_) due to the lost gp45:gp55 interaction.

(*Footnote *2. Another technical note: these effects are more readily noted with RNAP^T4 ^than with the unmodified *E. coli *RNAP, most probably because of the effect of modifying the αCTD after T4 infection: ADPribosylation at Arg265 in the DNA-binding helix of the αCTD eliminates or at least reduces DNA binding; DNA binding by the αCTD may interfere with gp45 access to gp33 more effectively in the case of "one-armed activation" (that is, activation by the sliding clamp connected to the RNAP holoenzyme only through the C-end of gp33) than in the case of bivalent attachment to the C-ends of both gp55 and gp33; ADPribosylation may eliminate or diminish the competition.)

Gp45 is the least stable of the sliding clamps [73,74] perhaps reflecting the fact that it is partly open in solution, and its DNA-tracking state is accordingly relatively transient [51,73]. This is proposed to be the mechanistic basis of the coupling of T4 late transcription to concurrent DNA replication *in vivo *[75]. The DNA-loading sites of sliding clamps are transient intermediates of replication: they are continuously created, predominantly by lagging strand DNA synthesis, and consumed as DNA discontinuities are sealed by ligation. Interrupting ongoing DNA replication quickly leads to a loss of clamp-loading sites, followed soon thereafter by a loss of DNA-loaded sliding clamps as they fall off DNA. This can be prevented if DNA ligation is also blocked and the resulting DNA breaks are stabilized against degradation--precisely the conditions under which T4 late gene expression becomes independent of DNA replication *in vivo*, as already described.

It is a common cellular strategy to make the expression of certain genes contingent on genome replication. Linking these separate processes involves symbolic communication provided by signaling pathways. Employing the DNA-loaded sliding clamp as the activator of T4 late transcription instead allows the state of DNA replication to be communicated directly through the availability of sliding clamp-loading sites, and dispenses with symbolically mediated signaling. One can think of the strategy as an instance of elegant streamlining or as a primitive relic.

### Phages of the T4 family

Sequenced genomes of T4-related phages (see [59], which is a review by Petrov, et al., in this series) infecting a wide range of bacterial hosts (*E. coli*, *Acinetobacter, Vibrio*, *Aeromonas*, marine cyanobacteria) permit a glance at the prevalence of the transcription system of which T4 is the prototype. Gene 45 and 55 homologues are members of the core gene set of this family of phages [76,77]. Strong conservation of amino acid sequence for extended segments of gp55, including its putative σ domain 2, have been commented on above; the hydrophobic C-terminal motif is also retained in gp55 homologues (Figure 2). Thus, it appears probable that a late transcription system based on gene 55 and the sliding clamp is a general feature of the multiplication cycles of the T4-related family of phages. Indeed highly similar consensus sequences have been identified (*in silico*) for *Vibrio *phage KVP40, *Aeromonas *phage 44RR, and the marine cyanophage S-PM2, and a closely related consensus (a/gC at positions -13/-12 in place of TA) has been found for the *Aeromonas *phage Aeh1 [77-79]. The role of gp33 homologues (Figure 4) is less obvious. Bivalent tethering of the late RNAP holoenzymes of the T4-related phages to their sliding clamps should suffice to generate activation by increasing the effective avidity of promoter binding. The coliphage gp33 homologues are identifiable as RNAP core- and sliding clamp-binding proteins and so are the *Aeromonas *phage homologues, with the exception of phage 65. Whether the two vibriophages, phage 65, and cyanobacterial phage SPM-2 homologues bind their conjugate sliding clamps is not made obvious by their sequences and consequently the mechanism of their participation in late transcription cannot be guessed by inspection.

Speculation about coupling of late transcription to concurrent DNA replication as a general feature of the multiplication cycles of these phages is on even shakier ground. Coupling is proposed to arise as a consequence of the instability of the DNA-mounted state of T4 gp45. The T4-related sliding clamps are all 3-domain PCNA-like rather than 2-domain bacterial type proteins, but whether they generally fall off DNA equally readily remains to be determined. Another feature of the T4 late transcription system is the high sequence similarity of the C-termini of gp43, gp55 and gp33 [69]. This is not a conserved feature of all the phages of this family. Thus, the sites of attachment of gp55, gp33 and DNA polymerase homologues to their conjugate sliding clamps may vary.

If gp55 and gp33 are primarily "merely" deviant σ domains 2 and 4, why are they invariably encoded by widely separated and separately regulated genes? Why is there no fused late-transcription σ? Some suggestions for why this hypothetical fusion protein does not exist in nature or, at any rate, has not been found, can be offered: 1) Physically separating these two domains weakens their competitive advantage for binding to RNAP core, and modulates the competition between middle and late transcription. If a hypothetical gp55-gp33 fusion protein has a great RNAP core-binding advantage over σ^70 ^and AsiA (the co-activator of T4 middle gene expression), then the dosage and timing of its production relative to the initiation of DNA replication become critical design elements of the viral multiplication cycle. In the extreme case, sufficiently premature and abundant production of the fusion protein might prevent DNA replication and shut down transcription. 2) The "split-σ" gp55/gp33 combination is a device for bivalent tethering of the sliding clamp to the late promoter, which optimizes late transcription. One way of approaching these questions is to design appropriate composite proteins and examine their modes of action and interaction *in vitro*. Experiments along those lines favor the first of these explanations and tend to discount the second: 1) Fused gp55-gp33 proteins with the gp55 sliding clamp-binding domain consequently internal instead of C-terminal are functional for sliding clamp-activated T4 late transcription so long as the length of the connector joining gp55 to the RNAP β flap-binding domain of gp33 is optimized. 2) The corresponding RNAP holoenzyme with its fused pseudo-σ subunit is almost completely inactive for basal transcription as a consequence of repression by its C-terminal gp33 domain. In that sense (essentially complete activator-dependence), the gp55-gp33 fusion version of the T4 late RNAP holoenzyme resembles σ^54^•RNAP. 3) When gp33 is covalently linked to gp55, suppression of basal transcription still depends on ability to bind to the β flap. 4) Fusing gp33 to gp55 generates an effective competitor against σ^70^•RNAP transcription at a strong -35/-10 type promoter [V. Jain & EPG, unpublished observations].

Coupling transcription of selected genes to specific states of the cell-division cycle, including S phase, is a ubiquitous strategy of cells and it ubiquitously engages signaling pathways, that is, molecular systems for generating messengers and interpreting messages. The mechanism that couples transcription of the viral late genes to replication in the T4 multiplication cycle elegantly dispenses with (or, depending on perspective, is too primitive for) symbolic communication, instead directly using universal components of cellular DNA replication, the primer-template junction and the clamp-loading factors, as generators of activation and the ubiquitous sliding clamp as the activator. It is puzzling that this efficient and direct regulatory device should be restricted to T4 and perhaps other members of the T4-related phage family. In fact, it has been possible to design a sliding clamp-activation domain fusion protein that generates clamp loader-dependent transcriptional activation of eukaryotic RNAP II *in vitro *[80]. Nevertheless, other instances of the use of this direct and simple mechanism for coupling transcriptional regulation to DNA replication in nature have not been found.

## Competing interests

The authors declare that they have no competing interests.

## Authors' contributions

EPG and GAK composed this review. Both authors have read and approved the final manuscript.
